# The Social Acceptance of Smart Health Services in Japan

**DOI:** 10.3390/ijerph19031298

**Published:** 2022-01-24

**Authors:** Yuho Shimizu, Aimi Ishizuna, Shin Osaki, Takaaki Hashimoto, Mitsuharu Tai, Tetsushi Tanibe, Kaori Karasawa

**Affiliations:** 1 Graduate School of Humanities and Sociology, The University of Tokyo, Tokyo 1130033, Japan; tanibe.tetsushi@gmail.com (T.T.); karasawa@l.u-tokyo.ac.jp (K.K.); 2 Faculty of Letters, The University of Tokyo, Tokyo 1130033, Japan; ishizuna-aimi@g.ecc.u-tokyo.ac.jp; 3 Graduate School of Frontier Sciences, The University of Tokyo, Tokyo 1138656, Japan; osaki@edu.k.u-tokyo.ac.jp; 4 Faculty of Sociology, Toyo University, Tokyo 1128606, Japan; hshmtsp@gmail.com; 5 Center for Technology Innovation, R&D Group, Hitachi, Ltd., Tokyo 1138656, Japan; mitsuharu.tai.wu@hitachi.com; 6 Center for Research and Development on Transition from Secondary to Higher Education, The University of Tokyo, Tokyo 1130033, Japan; 7 Faculty of Humanities, Niigata University, Niigata 9502181, Japan

**Keywords:** smart health, social acceptance, smart city, Japan, structural equation modeling

## Abstract

In recent years, smart health (s-Health) services have gained momentum worldwide. The s-Health services obtain personal information and aim to provide efficient health and medical services based on these data. In Japan, active efforts to implement these services have increased, but there is a lack of social acceptance. This study examined social acceptance concerning various factors such as trust in the city government, perceived benefits, perceived necessity, perceived risk, and concern about interventions for individuals. An online survey was conducted, and Japanese participants (*N* = 720) were presented with a vignette depicting a typical s-Health service overview. The results of structural equation modeling showed that trust was positively related to perceived benefit and necessity and negatively related to perceived risk and concern about interventions for individuals. Perceived benefit and trust were positively related to social acceptance, and perceived risk was negatively related to acceptance. The model obtained in this study can help implement s-Health services in public. Empirical studies that contribute to improving public health by investigating the social acceptance of s-Health services should be conducted in the future.

## 1. Introduction

### 1.1. Background and Purpose

In recent years, the implementation of smart cities has been accelerating worldwide, and the development of sustainable and environment-friendly cities using artificial intelligence (AI) and big data has been fostered [1,2,3]. In Japan, attention to these initiatives is also growing, and the Japan Cabinet Office aims to implement smart cities in more than 100 cities by 2025 [4]. One of the fundamental goals of smart city projects is to promote the health of all citizens [5,6]. As Lohachab showed, smart health (s-Health) service initiatives are being developed to provide efficient medical and health services by utilizing the networks and infrastructure of smart cities to achieve the above goal [7,8,9]. The implementation of s-Health services is gaining momentum worldwide [10,11]. In Japan, however, there are still no cities where s-Health services are in full operation. Nevertheless, initiatives are being conceived, planned, or partially tested to use AI to analyze big data, such as people’s health and medical data, prevent frailty among older adults [12], and improve the quality of healthcare services [13].

While these efforts have been active, significant issues remain to be addressed. One of these is the issue of social acceptance of s-Health services. In general, when new scientific technology is implemented in a particular region, there are scattered cases of citizens’ insufficient acceptance of the project [14,15]. An example of a project that became a social issue due to the strong opposition from the public is the Sidewalk Labs project in Toronto, Canada. A large amount of data was supposed to be collected and utilized in facilities throughout the city [16]. However, when it became explicit that the plan was to install sensors everywhere in the city and acquire personal information such as the location of smartphones, the project faced citizens’ strong opposition and eventually led to its cancellation [17]. In Japan, when Hamamatsu City in Shizuoka Prefecture solicited public comments about the “Hamamatsu City Digital Smart City Concept (draft),” there were many objections based on concerns about privacy protection [18]. In smart city projects, especially s-Health services, considerable personal information, including information on disease status, is acquired and utilized [7]. The damage caused to individuals by the leakage and abuse of such information is significant, and thus privacy protection is one of the greatest challenges in s-Health services [19,20,21]. The perceived risk of leakage and abuse of the obtained personal information may strongly influence social acceptance [22,23,24].

In this study, we focused on a specific aspect of s-Health services: the acquisition of detailed personal information. We summarized various factors, including perceived risk, which can influence the social acceptance of s-Health services. Specifically, we conducted an online survey of Japanese participants to clarify the relationship between each factor and presented a model of social acceptance. As the implementation of s-Health services has been active in Japan, it is essential to examine the social acceptance of the services.

### 1.2. Literature Review

We examine the determinants of the social acceptance of s-Health services by referring to a previous study recently conducted in Japan that dealt with the social acceptance of various smart city projects [25]. The study found that trust in business operators, perceived benefits, and perceived necessity were positively related to the social acceptance of the projects [25]. Many previous studies conducted in other countries have also suggested that trust and perceived benefit are critical factors in the social acceptance of smart cities [15,24,26]. In addition, it has been shown that people with higher trust have higher perceived benefit and necessity [25,26,27] and lower perceived risk [23,25,28]. This suggests that perceived benefit, necessity, and risk are determinants of the social acceptance of smart city projects. The above findings can also be applied to social acceptance of s-Health services, an initiative related to smart cities. We examine this issue in this study.

In contrast, there is a determinant of social acceptance that should be explicitly considered in the context of s-Health services. In these services, it has been noted that presenting the results obtained by analyzing personal data often directly influences people’s health behavior [29]. In a previous study conducted recently in Japan which summarized people’s expectations and concerns about health-related initiatives in smart cities [30], many concerns were expressed about the direct instructions for the individual’s health behavior based on personal health and medical data, namely, “concern about interventions for individuals,” such as “Being presented with risks related to one’s health and diseases increases anxiety” [30]. It has been noted that personal health and medical information is one of the most private types of data [31,32]. There are also likely to be many people who feel that s-Health services are provided in a surveillance society [33,34]. Therefore, concern about interventions for individuals may be related to the social acceptance of s-Health services, which this study examines.

### 1.3. Overview

Based on the above findings, this study presented participants with a vignette depicting a plan of the specific s-Health service that measured social acceptance, trust in the city government, perceived benefit, perceived necessity, perceived risk, and concern about interventions for individuals. The vignette was developed by referring to the case of Aizuwakamatsu City in Fukushima Prefecture in Japan and Kashiwa City in Chiba Prefecture in Japan, where s-Health services have been conceived, planned, or partially tested [12,13]. In addition, previous studies on perceived risk dealt with two separate variables [22,35]: the probability of risks (the likelihood of a particularly damaging event occurring) and the size of risks (the degree of damage that would be incurred if it occurred). Hence, this study distinguished between them and examined the effect of perceived risk in detail. Referring to the previous study, which was conducted recently in Japan and dealt with the social acceptance of an extensive range of smart city projects [25], we examined a model in which trust in the city government influenced social acceptance through the mediation of perceived benefit, perceived necessity, perceived risk, and concern about interventions for individuals.

The novelty of this study is that we applied the findings of previous studies [15,24,25,26,27,28,30] to s-Health services and verified its validity in this matter. Specifically, we presented a model of the social acceptance of s-Health services that incorporated several factors: trust in the city government, perceived benefit, perceived necessity, perceived risk, and concern about interventions for individuals. There is an urgent need to increase social acceptance to facilitate s-Health services, which are expected to become more prevalent worldwide, including Japan. This study has theoretical significance in presenting the model of social acceptance and the practical significance of clarifying the aspects that local governments and companies should pay attention to when promoting s-Health services to the general public. Implementing the findings on social acceptance of a broad range of smart cities to each specific context, including s-Health services, will help identify the characteristics of each project. The need for empirical studies, such as this study, will increase as various initiatives are undertaken in smart cities.

## 2. Materials and Methods

### 2.1. Participants

We recruited participants through CrowdWorks, a crowdsourcing service. CrowdWorks has the largest number of registered users in Japan [36], and the participants in this study aligned in demographic composition with the registered users. A total of 720 Japanese individuals (280 males and 440 females, 19–73 years old) participated in this study. The mean age of the participants was 39.48 years old (*SD* = 10.87). This study was approved by the Ethical Committee of the University of Tokyo and was conducted in October 2021.

### 2.2. Vignette

We referred to the case of Aizuwakamatsu City in Fukushima Prefecture in Japan and Kashiwa City in Chiba Prefecture in Japan, where s-Health services are being conceived, planned, or partially tested [12,13]. The vignette depicted a service that collects citizens’ health and medical data and suggests preventive measures against diseases. Specifically, the vignette included descriptions of the content of collected data, management entities, collection methods, and utilization methods. In addition, in line with the actual service plan, it was clearly stated that those who do not wish to provide data could refuse. The complete vignette can be found in Appendix A.

### 2.3. Items

Except for demographic items (age, gender, and nationality), all items were asked using a six-point Likert scale, with higher scores indicating a higher degree. All questionnaire items were provided in supplementary materials in the Open Science Framework (OSF) repository (https://osf.io/xch5a/?view_only=68df0dea4a4146a7966798443aa9c594, 17 January 2022).

Social acceptance was measured using the three items (α = 0.89; e.g., “Assuming you live in City A, and you want to help with this project as a resident”), and the mean was scored [25,27]. Trust in the city government was measured using a single item (“You trust City A to implement this project”) [15,25]. The perceived benefit was measured using the two items of benefit for self (*r* = 0.74, *p* < 0.001; e.g., “Assuming you live in City A, this project will have a positive impact on you”) and the two items of benefit for whole citizens (*r* = 0.52, *p* < 0.001; e.g., “This project will have a positive impact on all the citizens”), and the mean was scored [25,27]. Perceived necessity was measured using two items (*r* = 0.66, *p* < 0.001; e.g., “This project is highly necessary for you”), and the mean was scored [25]. Perceived risk was measured using a single item of the probability of risks (“When you provide personal information for this project, there is a high probability of personal information being leaked”) and a single item of the size of risks (“If personal information provided for this project is leaked, the damage to people will be great”) [22,25,37]. With reference from a previous study [30], concern about interventions for individuals was measured using a single item (“The individualized prediction of health risks will increase my anxiety”). In addition, participants’ perceived health competence was measured using the Japanese version of the Perceived Health Competence Scale [38] as the control variable. The scale consisted of eight items (α = 0.90; e.g., “I handle myself well with respect to my health”), and the mean was scored.

### 2.4. Procedure and Analysis

The survey was conducted online. Participants were briefed on the study, agreed to participate, and read the vignette described above. We did not set a time limit for reading the vignette and asked the participants to move forward when they fully understood the contents. Subsequently, they responded to the four items, confirming that they had read the vignette correctly (see OSF). Data from participants who answered even one of these incorrectly were not used in the analysis. The participants responded to the items of social acceptance, trust in the city government, perceived benefit, perceived necessity, perceived risk, concern about interventions for individuals, perceived health competence, and demographics.

The statistical software R (ver. 4.1.0, R Foundation for Statistical Computing, Vienna, Austria) was used for the analysis. The statistical significance level was set to α = 0.05. No missing values were found in the data obtained in this study. The data and R scripts used in the analysis were posted on the OSF.

## 3. Results

### 3.1. Data Screening

Participants who answered incorrectly to the item “Please select ‘agree’ for this item” (*N* = 41) and even one of the four items to confirm that they had read the vignette correctly (*N* = 49) were excluded from the analysis (including duplicates). As a result of the screening, the data used in the analysis were *N* = 636 (19–72 years), with a mean age of 39.74 years (*SD* = 10.84) and 248 males and 388 females. The means, standard deviations, and correlation coefficients for each variable are listed in Table 1. The correlation coefficients between the variables were all significant. The absolute values of the correlation coefficients between the majority of the variables were above 0.30, suggesting a medium effect size [39]. Even the smallest absolute value of the correlation coefficient was above 0.10, indicating a small effect size [39]. The analysis results using the data of all participants without screening (*N* = 720) were posted on the OSF. No remarkable difference from the results reported in the main text below was found.

### 3.2. Structural Equation Modeling

Structural equation modeling (SEM) was conducted to examine a model wherein trust in the city government influences social acceptance through the mediation of perceived benefit, perceived necessity, perceived risk, and concern about interventions for individuals (Model 1). The perceived benefit was a latent variable consisting of benefit for self and benefit for whole citizens, and perceived risk was a latent variable consisting of the probability of risks and the size of risks, respectively. We controlled the participants’ perceived health competence, age, and gender, assuming simple covariates in the model. We also conducted SEM without control variables, and the results were not significantly different from those reported in this paper (see OSF). Furthermore, the results for participants with high/low perceived health competence, older/younger participants, and male/female participants were posted on the OSF.

**Figure 1:**
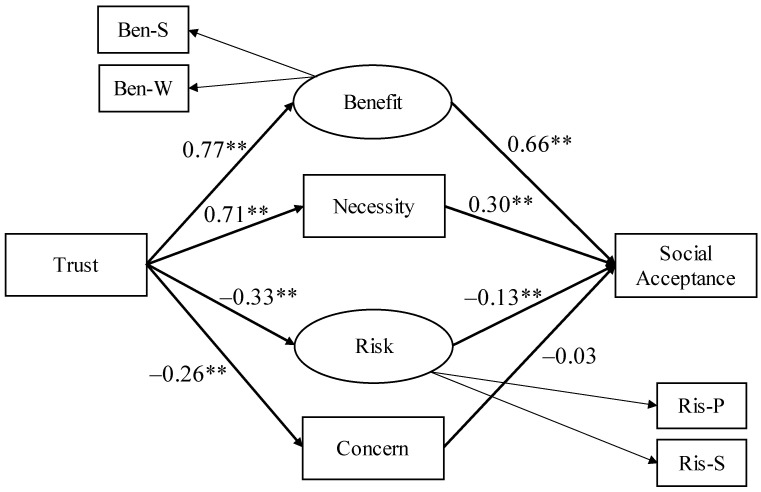
Results of Model 1. Coefficients are standardized and all coefficients of measurement equations are significant (β > 0.64, *p* < 0.001). Ben-S: Benefit for self, Ben-W: Benefit for whole citizens, Ris-P: Probability of risks, Ris-S: Size of risks. ** *p* < 0.01.

**Figure 2:**
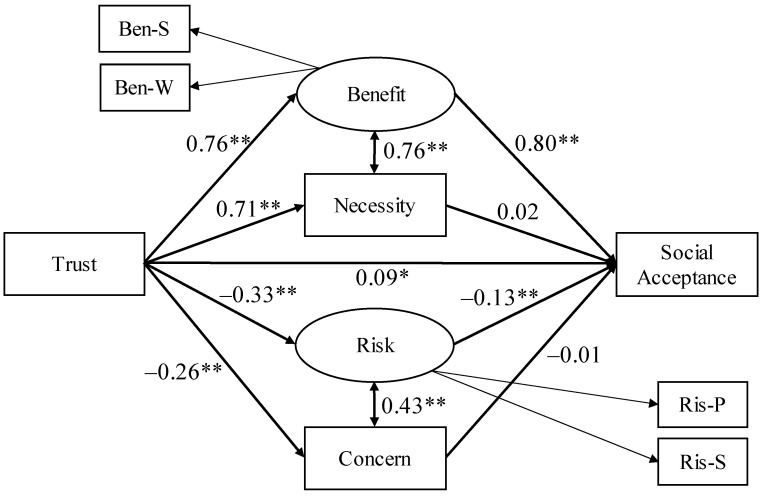
Results of Model 2. Coefficients are standardized and all coefficients of measurement equations are significant (β > 0.67, *p* < 0.001). * *p* < 0.05, ** *p* < 0.01.

The goodness of fit for Model 1 was not satisfactory, with RMSEA = 0.21, AGFI = 0.45, and CFI = 0.86 (Figure 1). Later, referring to a previous study [25], we included that trust in the city government directly predicted social acceptance. We also added the covariate relationship between perceived benefit and necessity and between perceived risk and concern about interventions for individuals (Model 2), referring to the correlation coefficients between the variables (Table 1). The goodness of fit for Model 2 was determined to be satisfactory, with RMSEA = 0.03, AGFI = 0.96, and CFI > 0.99 (Figure 2). We also analyzed the model using Bayesian SEM, and the results were not significantly different from those reported in this paper (see OSF). In addition, we investigated the mediation effects of perceived benefit, perceived necessity, perceived risk, and concern about interventions for individuals, and the results were posted on the OSF.

## 4. Discussion

In this study, we presented the participants with a vignette depicting a plan for particularly s-Health service and examined the model of social acceptance. As a result, the obtained model showed satisfactory goodness of fit, with those who had higher trust in the city government having higher perceived benefit and necessity and lower perceived risk and concern about interventions for individuals. Perceived benefit and trust in the city government were positively related to social acceptance, and perceived risk was negatively related to social acceptance. In summary, the findings of previous studies [15,24,25,26,27,28,30] were shown to be valid for s-health services.

### 4.1. Various Factors Related to the Social Acceptance of s-Health Services

Perceived benefit and trust in the city government were positively related to social acceptance of s-Health services. This result is consistent with many previous studies dealing with social acceptance of smart city projects [15,24,25,26,27]. In particular, there was a significant relationship between perceived benefit and social acceptance, which can be attributed to the s-Health service depicted in our vignette being more closely associated with the individuals’ lives than other kinds of smart city projects and being directly related to their health behaviors. In addition, personal health and medical information collected in s-Health services are highly private data [31,32]. Since people tend to make more elaborate decisions about the targets closely related to themselves [40,41], the participants were believed to make more elaborate information processing about the extent of future benefits from the service depicted in the vignette. This may have led to a substantial relationship between perceived benefits and social acceptance. Considering the above, it is essential to increase perceived benefits to enhance social acceptance of s-Health services.

In contrast, the direct effect of trust in the city government on social acceptance was relatively small, but there was a significant mediation effect of perceived benefit (see OSF). Referring to the research on persuasion in social psychology, trust in the other person is a peripheral cue, and deciding on the target based on trust alone is regarded as information processing through peripheral routes [42,43,44]. As mentioned above, compared to other kinds of smart city projects, s-Health services are more closely related to the lives of general citizens and the issue of privacy protection [31,32]. Hence, in this study, elaborate information processing regarding future benefits seemed to be more active than information processing regarding trust in the city government, the peripheral cue.

Perceived risk was negatively related to social acceptance of s-Health services. Since detailed personal information about an individual’s health and medical data is captured and utilized in s-Health services, privacy protection is a crucial concern [19,20,21]. Therefore, the perceived risk of personal information leakage significantly impacts social acceptance [22,23,24]. In addition, perceived risk scores in this study were remarkably higher than those in the previous study [25], which was recently conducted in Japan and dealt with the social acceptance of various smart city projects. This suggests that the risk of leakage of highly private data, especially those collected in s-Health services, is likely to be strongly perceived [31,32]. In contrast, concerns about interventions for individuals did not have a significant relationship with social acceptance (Figure 2). However, the significant covariate relationship between concern about interventions for individuals and perceived risk suggests that concerns are essential variables when examining the social acceptance of s-Health services. The concept of concern about interventions for individuals has not been adequately addressed in previous studies and needs to be examined in more detail, including the elaboration of the concept.

### 4.2. Limitations

Although the above findings were obtained in this study, the following limitations exist. First, the factors discussed as necessary in previous studies were not exhaustively examined in this study. For example, we did not examine the relationship between perceived fairness and social acceptance of s-Health services. Previous research on social acceptance of wind power facilities [15,45,46], hydropower facilities [47], and high-level radioactive waste management [48] showed that distributive justice, which is perceived fairness in distributional outcomes, and procedural justice, which is perceived fairness in distributional procedures, each influences social acceptance. In s-Health services, the impact of perceived fairness deserves consideration because those who benefit substantially from the services may be limited to older adults and people at relatively high risk for illnesses. In addition, given the reported significant relationship between perceived fairness and trust [49,50], the relationship between perceived fairness and the social acceptance of s-Health services should be examined in detail. It is necessary to extend the model obtained in this study by referring to various previous studies that have examined the social acceptance of a wide range of scientific technologies.

Second, we conducted an online survey using CrowdWorks. We did not use random sampling to measure specific aspects of the participants’ demographics (e.g., social status, marital status, educational level) because it is a problem of online surveys that collecting such personal information increases the number of participants who do not want to participate in the survey, thus increasing the sampling bias. However, the influence of each variable mentioned above should be examined in future studies.

Third, we conducted a survey and analyzed the data. These are not longitudinal or experimental data; accordingly, it is difficult to conclude that our model is the most appropriate. The findings of this study are consistent with the results of previous studies [15,23,24,25,26,27,28,29,30,31,32,33,34] and the empirical findings related to the technology acceptance model, which deals with the social acceptance of scientific technology in general [51,52]. However, for a survey such as the one conducted in this study, the inability to sufficiently evaluate causal relationships is a significant and universal problem. We should overcome this problem by including experimental manipulations in the future.

## 5. Conclusions

We presented a model of social acceptance by applying the findings of previous studies [15,24,25,26,27,28,30] to s-Health services. In future research, the possible impact of common method bias on the results should be examined [53]. For example, participants may speculate that researchers are asking for positive feedback on the vignette presented to the participants. In addition, although our survey is unlikely to include sensitive content, social desirability tendencies may need to be considered.

As Chadborn et al. suggested, a weakness of s-Health services is that older adults and other people prone to illness are the primary users of the services, and these groups often have difficulty using the Internet and smartphones, which are the means of providing these services [54]. A service that is favorably received by the general public may not be fully accepted by older adults or those with chronic diseases [55]. Therefore, the future challenge is to provide s-Health services to such people through user-friendly technologies [56]. Achieving this aim will lead to an increase in the social acceptance of s-Health services.

In this study, the results showed that perceived benefit, trust in the city government, and perceived risk significantly influenced social acceptance, contributing to the practical efforts to enhance citizens’ acceptance of the services. For example, it is vital for local governments and companies promoting s-Health services to patiently appeal to citizens that the effective use of personal health and medical data can provide significant benefits beyond conventional services. It is also necessary for providers to actively create opportunities to consult with the public and foster trust [14]. There is a need for more empirical studies that can improve public health by increasing the social acceptance of s-Health services. Implementing s-Health services is expected to occur in full swing in Japan. Therefore, we should conduct a longitudinal study on the extent to which social acceptance will change after services that acquire and utilize a large amount of personal information are launched.

## Figures and Tables

**Table 1:** Means, standard deviations, and correlation coefficients for each variable.

		*M*	*SD*	1	2	3	4	5	6	7
1	Soc-A	4.02	0.96	―						
2	Tru	3.71	0.92	0.75	―					
3	Ben-S	4.13	0.96	0.84	0.67	―				
4	Ben-W	4.17	0.86	0.75	0.64	0.73	―			
5	Nec	3.81	0.96	0.83	0.71	0.80	0.72	―		
6	Ris-P	4.11	1.11	−0.33	−0.26	−0.24	−0.20	−0.27	―	
7	Ris-S	4.57	1.20	−0.26	−0.20	−0.15	−0.15	−0.19	0.51	―
8	Con	3.42	1.14	−0.31	−0.26	−0.25	−0.23	−0.26	0.35	0.33

Soc-A: Social acceptance, Tru: Trust in the city government, Ben-S: Benefit for self, Ben-W: Benefit for whole citizens, Nec: Necessity, Ris-P: Probability of risks, Ris-S: Size of risks, Con: Concern about interventions for individuals. All correlation coefficients were *p* < 0.001.

## Data Availability

The data presented in this study are openly available in the OSF supplementary materials.

## Supplementary Materials

The following are available online at https://www.mdpi.com/article/10.3390/ijerph19031298/s1.

## Appendix A. Vignette

As a part of the smart city plan, City A is planning an Information and Communication Technology (ICT)-based health promotion service. This service will first collect health data from citizens and then propose preventive measures against diseases according to the health status of each individual.

The health data to be collected includes information on the citizens’ health checkups, hospital visits, medication history, and lifestyle data such as exercise status and vital data (e.g., pulse rate, blood pressure, and body temperature) obtained through smartphone applications. The collected data will be managed by the local government.

By analyzing the collected health data and predicting the health risks of the citizens individually, preventive measures against diseases tailored to each individual will be proposed to the citizens through smartphone applications. The collected health data will also be used to implement medical services that meet the needs of the citizens. Improving the health of citizens is expected to reduce the cost of medical care and welfare.

In addition, data held by medical institutions (data on the citizens’ health checkups, hospital visits, and medication history) are allowed to be used in this plan by the council, which consists of experts and representatives of the citizens. The process is based on the law, but individuals who do not wish to provide their data can refuse. In addition, the city will obtain the consent of individuals when providing the data and will not collect data from individuals whose consent cannot be obtained.

## References

[1] Granier B., Kudo H. (2016). How are citizens involved in smart cities? Analysing citizen participation in Japanese “Smart Communities”. Sci. Inf. Policy.

[2] Khansari N., Mostashari A., Mansouri M. (2013). Impacting sustainable behavior and planning in smart city. Int. J. Sustain. Land Use Urban Plan..

[3] Neirotti P., De Marco A., Cagliano A.C., Mangano G., Scorrano F. (2014). Current trends in Smart City initiatives: Some stylised facts. Cities.

[4] Japan Cabinet Office (2021). Society 5.0. https://www8.cao.go.jp/cstp/society5_0/index.html.

[5] Al-Azzam M., Alazzam M.B. (2019). Smart city and smart-health framework, challenges and opportunities. Int. J. Adv. Comput. Sci. Appl..

[6] Trencher G., Karvonen A. (2019). Stretching “smart”: Advancing health and well-being through the smart city agenda. Local Environ..

[7] Lohachab A. (2022). Bootstrapping urban planning: Addressing big data issues in smart cities. Research Anthology on Big Data Analytics, Architectures, and Applications.

[8] Sicari S., Rizzardi A., Grieco L.A., Piro G., Coen-Porisini A. (2017). A policy enforcement framework for Internet of Things applications in the smart health. Smart Health.

[9] Solanas A., Patsakis C., Conti M., Vlachos I.S., Ramos V., Falcone F., Postolache O., Perez-Martinez P.A., Pietro R.D., Perrea D.N. (2014). Smart health: A context-aware health paradigm within smart cities. IEEE Commun. Mag..

[10] Abdellatif A.A., Mohamed A., Chiasserini C.F., Tlili M., Erbad A. (2019). Edge computing for smart health: Context-aware approaches, opportunities, and challenges. IEEE Netw..

[11] Ahad A., Tahir M., Aman Sheikh M., Ahmed K.I., Mughees A., Numani A. (2020). Technologies trend towards 5G network for smart health-care using IoT: A review. Sensors.

[12] Kashiwanoha Smart City (2020). Kashiwanoha Smart City: Action Plan. https://www.kashiwanoha-smartcity.com/images/pdf/kashiwanoha-smartcity-action-plan.pdf.

[13] Japan Ministry of Internal Affairs and Communications (2017). Supplementary Budget for IoT Service Creation: Result Report in 2015. https://www.soumu.go.jp/midika-iot/admin/wp-content/uploads/2016/07/H27-4_Report.pdf.

[14] Soma K., Haggett C. (2015). Enhancing social acceptance in marine governance in Europe. Ocean Coast. Manag..

[15] Sonnberger M., Ruddat M. (2017). Local and socio-political acceptance of wind farms in Germany. Technol. Soc..

[16] Tenney M., Garnett R., Wylie B. (2020). A theatre of machines: Automata circuses and digital bread in the smart city of Toronto. Can. Geogr..

[17] Keymolen E., Voorwinden A. (2020). Can we negotiate? Trust and the rule of law in the smart city paradigm. Int. Rev. Law Comput. Technol..

[18] Hamamatsu City (2021). Results of public comment on the Hamamatsu City Digital Smart City Concept (Draft). https://www.city.hamamatsu.shizuoka.jp/dsc/dejisuma_kousou/kangaekata/top.html.

[19] Ding D., Conti M., Solanas A. A smart health application and its related privacy issues. Proceedings of the 2016 Smart City Security and Privacy Workshop (SCSP-W).

[20] Liu H., Yao X., Yang T., Ning H. (2018). Cooperative privacy preservation for wearable devices in hybrid computing-based smart health. IEEE Internet Things J..

[21] Zhang Y., Zheng D., Deng R.H. (2018). Security and privacy in smart health: Efficient policy-hiding attribute-based access control. IEEE Internet Things J..

[22] Gerber N., Reinheimer B., Volkamer M. (2019). Investigating people’s privacy risk perception. Proc. Priv. Enhancing Technol..

[23] Wang J., Zhao S., Zhang W., Evans R. (2021). Why people adopt smart transportation services: An integrated model of TAM, trust and perceived risk. Transp. Plan. Technol..

[24] Wiegard R.B., Breitner M.H. (2019). Smart services in healthcare: A risk-benefit-analysis of pay-as-you-live services from customer perspective in Germany. Electron. Mark..

[25] Shimizu Y., Osaki S., Hashimoto T., Karasawa K. (2021). The social acceptance of collecting and utilizing personal information in smart cities. Sustainability.

[26] Choi J.K., Ji Y.G. (2015). Investigating the importance of trust on adopting an autonomous vehicle. Int. J. Hum.-Comput. Interact..

[27] Hashimoto T., Tham Y.J., Karasawa K., Tai M. Understanding people’s attitudes toward a “data-driven” society based on trust and technology acceptance models. Proceedings of the 84th Annual Convention Japanese Psychological Association.

[28] Neupane C., Wibowo S., Grandhi S., Deng H. (2021). A trust-based model for the adoption of smart city technologies in Australian regional cities. Sustainability.

[29] Hitachi and U-Tokyo Joint Research (2018). Society 5.0: A Human-Centered, Super-Smart Society.

[30] Shimizu Y., Osaki S., Hashimoto T., Karasawa K. (2021). How do people view various kinds of smart city services? Focus on the acquisition of personal information. Sustainability.

[31] Oh S.R., Seo Y.D., Lee E., Kim Y.G. (2021). A comprehensive survey on security and privacy for electronic health data. Int. J. Environ. Res. Public Health.

[32] Xiang D., Cai W. (2021). Privacy protection and secondary use of health data: Strategies and methods. BioMed Res. Int..

[33] Mani Z., Chouk I. (2019). Impact of privacy concerns on resistance to smart services: Does the ‘Big Brother effect’ matter?. J. Mark. Manag..

[34] Panchatcharam P., Vivekanandan S. (2019). Internet of things (IOT) in healthcare: Smart health and surveillance, architectures, security analysis and data transfer: A review. Int. J. Softw. Innov..

[35] Beck K.H. (1984). The effects of risk probability, outcome severity, efficacy of protection and access to protection on decision making: A further test of protection motivation theory. Soc. Behav. Pers..

[36] CrowdWorks (2021). One of the Largest Crowdsourcing Services. https://crowdworks.jp/.

[37] Krasnova H., Veltri N.F. Privacy calculus on social networking sites: Explorative evidence from Germany and USA. Proceedings of the 2010 43rd Hawaii International Conference on System Sciences.

[38] Togari T., Yamazaki Y., Koide S., Miyata A. (2006). Reliability and validity of the modified Perceived Health Competence Scale (PHCS) Japanese version. Jpn. J. Public Health.

[39] Cohen J. (1992). A power primer. Psychol. Bull..

[40] Chen S., Duckworth K., Chaiken S. (1999). Motivated heuristic and systematic processing. Psychol. Inq..

[41] Katz S.J., Erkkinen M., Lindgren B., Hatsukami D. (2018). Assessing the impact of conflicting health warning information on intentions to use e-cigarettes: An application of the Heuristic-Systematic model. J. Health Commun..

[42] Martin S.S., Camarero C., José R.S. (2011). Does involvement matter in online shopping satisfaction and trust?. Psychol. Mark..

[43] Yang S.C., Hung W.C., Sung K., Farn C.K. (2006). Investigating initial trust toward e-tailers from the elaboration likelihood model perspective. Psychol. Mark..

[44] Yen Y.S. (2018). Route factors influencing trust and attitude toward TV shopping. Serv. Ind. J..

[45] Firestone J., Hirt C., Bidwell D., Gardner M., Dwyer J. (2020). Faring well in offshore wind power siting? Trust, engagement and process fairness in the United States. Energy Res. Soc. Sci..

[46] Liebe U., Bartczak A., Meyerhoff J. (2017). A turbine is not only a turbine: The role of social context and fairness characteristics for the local acceptance of wind power. Energy Policy.

[47] Tabi A., Wüstenhagen R. (2017). Keep it local and fish-friendly: Social acceptance of hydropower projects in Switzerland. Renew. Sustain. Energy Rev..

[48] Choi Y., Matsuoka S. (2020). The relationship between trust, procedural justice, and distributive justice in high-level radioactive waste (HLW) management. J. Environ. Inf. Sci..

[49] Goedkoop F., Devine-Wright P. (2016). Partnership or placation? The role of trust and justice in the shared ownership of renewable energy projects. Energy Res. Soc. Sci..

[50] Huijts N.M., Molin E.J., Steg L. (2012). Psychological factors influencing sustainable energy technology acceptance: A review-based comprehensive framework. Renew. Sustain. Energy Rev..

[51] Davis F.D., Bagozzi R.P., Warshaw P.R. (1989). User acceptance of computer technology: A comparison of two theoretical models. Manag. Sci..

[52] Ghazizadeh M., Lee J.D., Boyle L.N. (2012). Extending the technology acceptance model to assess automation. Cogn. Technol. Work..

[53] Podsakoff P.M., MacKenzie S.B., Lee J.Y., Podsakoff N.P. (2003). Common method biases in behavioral research: A critical review of the literature and recommended remedies. J. Appl. Psychol..

[54] Chadborn N.H., Blair K., Creswick H., Hughes N., Dowthwaite L., Adenekan O., Pérez Vallejos E. (2019). Citizens’ juries: When older adults deliberate on the benefits and risks of smart health and smart homes. Healthcare.

[55] Ebihara J., Nakamura S. (2019). Smart City 5.0: Urban Operating Systems to Accelerate Regional Development.

[56] Jovanović M., De Angeli A., McNeill A., Coventry L. (2021). User requirements for inclusive technology for older adults. Int. J. Hum.-Comput. Interact..

