# Supplementary material for: The Social Acceptance of Smart Health Services in Japan
Source: Int J Environ Res Public Health. 2022 Jan 24;19(3):1298. doi: 10.3390/ijerph19031298 (PMC8834830; doi:10.3390/ijerph19031298)
Supplement: Supplementary file 1 [file ijerph-19-01298-s001.zip › ijerph-1541745-supplementary.pdf]

Supplementary Materials

1. Items

(1. strongly disagree, 2. disagree, 3. slightly disagree, 4. slightly agree, 5. agree, 6. strongly agree)

The (R) means reverse-scored.

1. **Social Acceptance**
   - Assuming you live in City A, and you want to help with this project as a resident.
   - This kind of project is a good idea.
   - You feel positive about this kind of project.

- **Trust in the City Government**
  - You trust City A to implement this project.

- **Benefit for Self**
  - Assuming you live in City A, this project will have a positive impact on you.
  - Assuming you live in City A, this project will help you stay healthy.

- **Benefit for Whole Citizens**
  - This project will have a positive impact on all the citizens.
  - This project will help reduce healthcare costs for society as a whole.

- **Necessity**
  - This project is highly necessary for you.
  - This project is highly necessary for society as a whole.

- **Probability of Risks**
  - When you provide personal information for this project, there is a high probability of personal information being leaked.

- **Size of Risks**
  - If personal information provided for this project is leaked, the damage to people will be great.

- **Concern About Interventions for Individuals**
  - The individualized prediction of health risks will increase my anxiety.

- **Perceived Health Competence**
  - I handle myself well with respect to my health.
  - No matter how hard I try, my health just does not turn out the way I would like. (R)
  - It is difficult for me to find effective solutions to the health problems that come my way. (R)
  - I succeed in the projects I undertake to improve my health.
  - I am gradually able to accomplish my goals with respect to my health.
  - I find my efforts to change things I do not like about my health are ineffective. (R)
  - Typically, my plans for my health do not work out well. (R)
  - I am able to do things for my health as well as most other people.

- **Check to See If Participants Read the Vignette Correctly**

  The participants answered "yes" if the following sentences matched the vignette and "no" if they did not.

  - City A is planning a health promotion service. (answer = yes)
  - In this project, health data will be collected and analyzed to help suggest disease prevention measures to individuals. (answer = yes)
  - Individuals who do not wish to be provided with data held by a medical institution can reuse to do so. (answer = yes)
  - The data collected from the applications will take the consent of individuals when providing the data. (answer = yes)

2. Structural Equation Modeling Without Data Screening

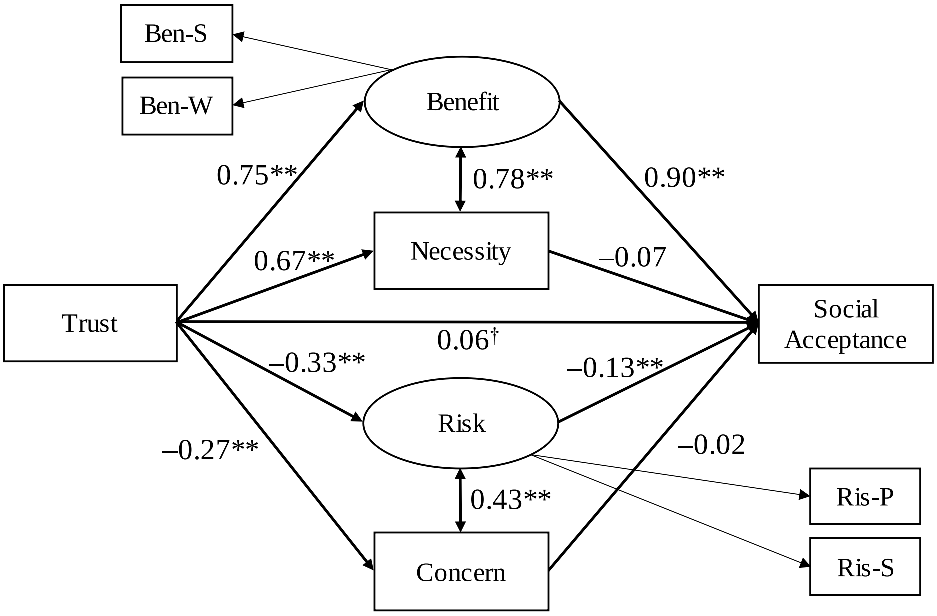

Figure S1. Results of SEM without data screening (N = 720). RMSEA = 0.04, AGFI = 0.95, CFI > 0.99. Coefficients are standardized and all coefficients of measurement equations are significant ($\beta > 0.65$, $p < 0.001$). Ben-S: Benefit for self, Ben-W: Benefit for whole citizens, Ris-P: Probability of risks, Ris-S: Size of risks. † $p < 0.10$, ** $p < 0.01$.

3. Structural Equation Modeling Without Control Variables

We also conducted SEM without control variables (Figure S2). Even when we did not control for these variables, the results were not significantly different from those reported in the main text.

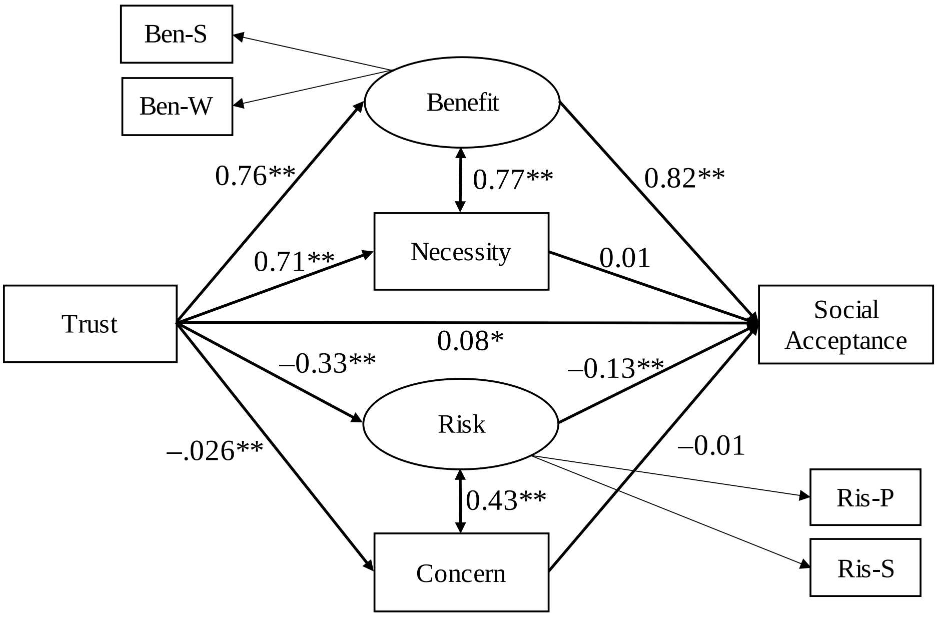

Figure S2. Results of SEM without control variables. RMSEA = 0.04, AGFI = 0.98, CFI > 0.99. Coefficients are standardized and all coefficients of measurement equations are significant ($\beta > 0.67$, $p < 0.001$). * $p < 0.05$, ** $p < 0.01$.

4. Structural Equation Modeling on Each Group Based on the Control Variables

We also conducted SEM on the data of each group, based on the perceived health competence, age, and gender of the participants. The results of participants with high perceived health competence (Figure S3), with low perceived health competence (Figure S4), with older participants (Figure S5), with younger participants (Figure S6),

with male participants (Figure S7), and with female participants (Figure S8) are shown below, respectively. Regarding the participants' perceived health competence and age, each mean was used as a boundary for grouping. For all subgroups, the results were not significantly different from those reported in the main text.

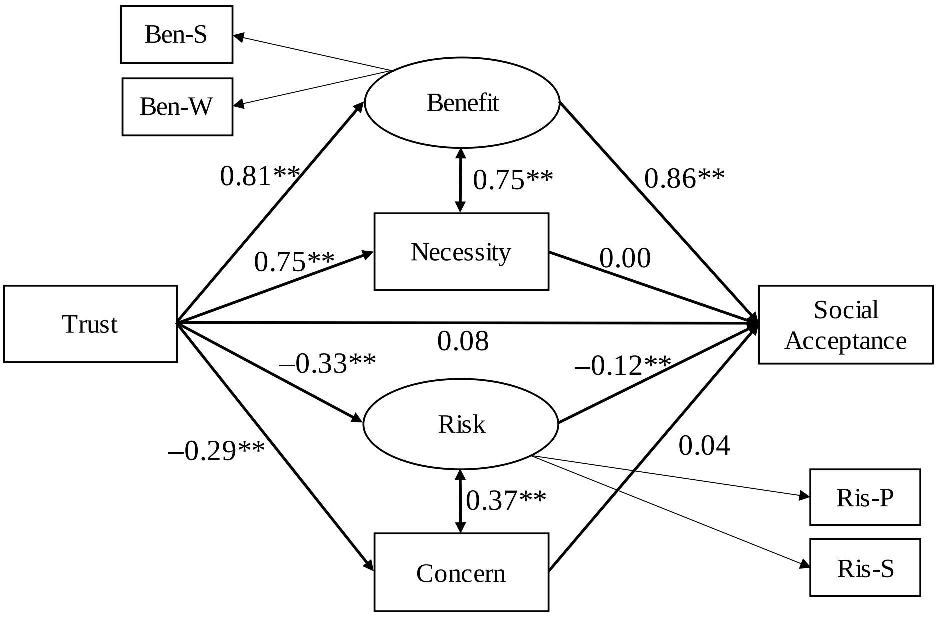

Figure S3. Results of SEM on the participants with high perceived health competence (N = 301). RMSEA = 0.07, AGFI = 0.90, CFI = 0.99. Coefficients are standardized and all coefficients of measurement equations are significant ($\beta > 0.71$, $p < 0.001$). ** $p < 0.01$.

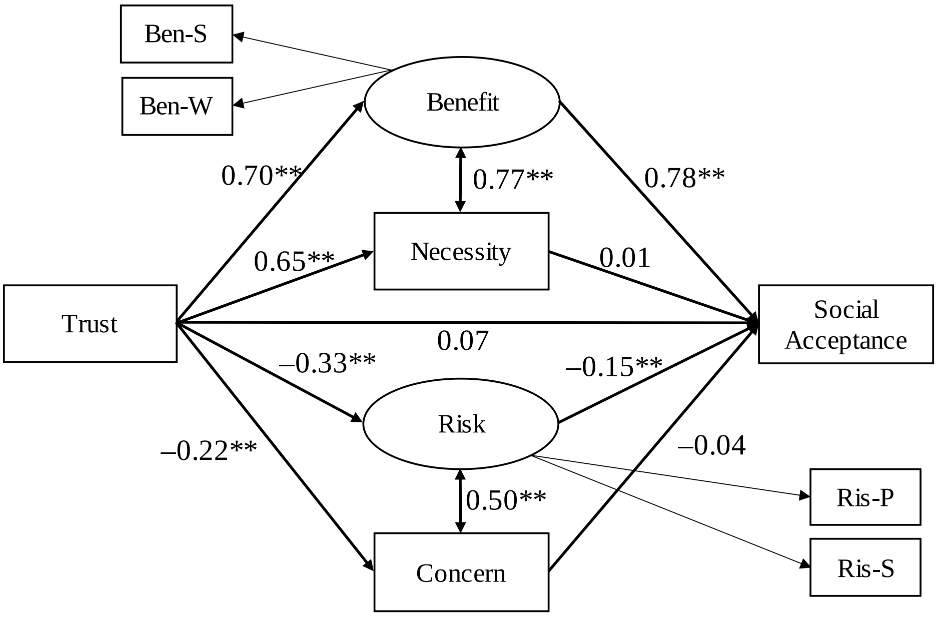

Figure S4. Results of SEM on the participants with low perceived health competence (N = 335). RMSEA = 0.02, AGFI = 0.95, CFI > 0.99. Coefficients are standardized and all coefficients of measurement equations are significant ($\beta > 0.63$, $p < 0.001$). ** $p < 0.01$.

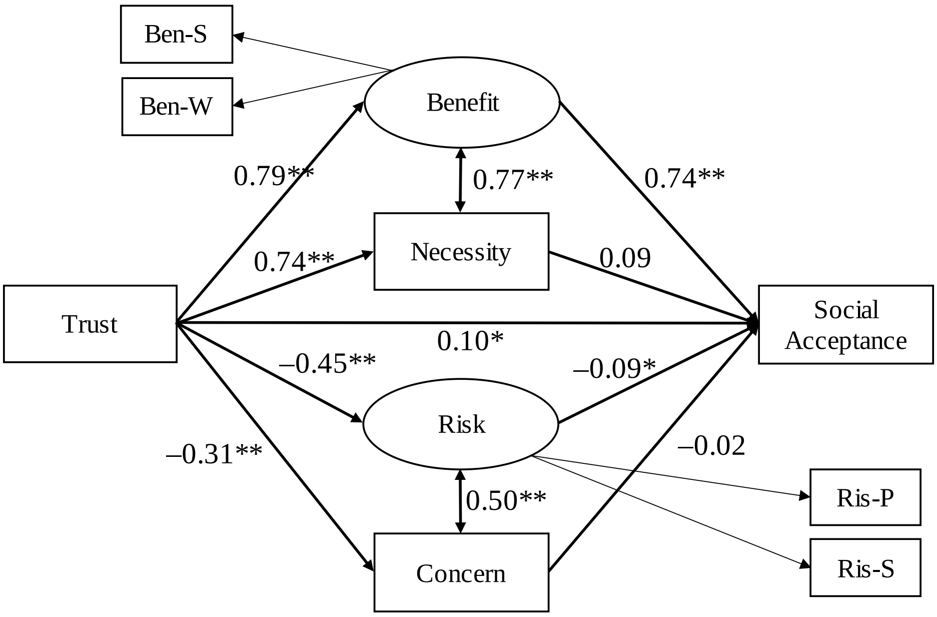

Figure S5. Results of SEM on older participants (N = 298). RMSEA = 0.05, AGFI = 0.93, CFI > 0.99. Coefficients are standardized and all coefficients of measurement equations are significant ($\beta > 0.69$, $p < 0.001$). * $p < 0.05$, ** $p < 0.01$.

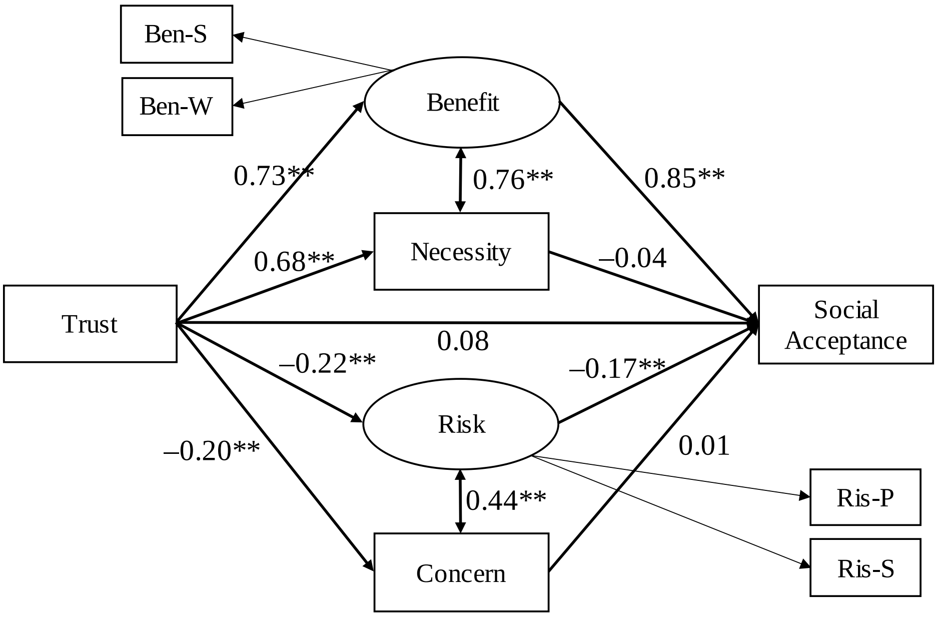

Figure S6. Results of SEM on younger participants (N = 338). RMSEA = 0.00, AGFI = 0.97, CFI > 0.99. Coefficients are standardized and all coefficients of measurement equations are significant ($\beta > 0.65$, $p < 0.001$). ** $p < 0.01$.

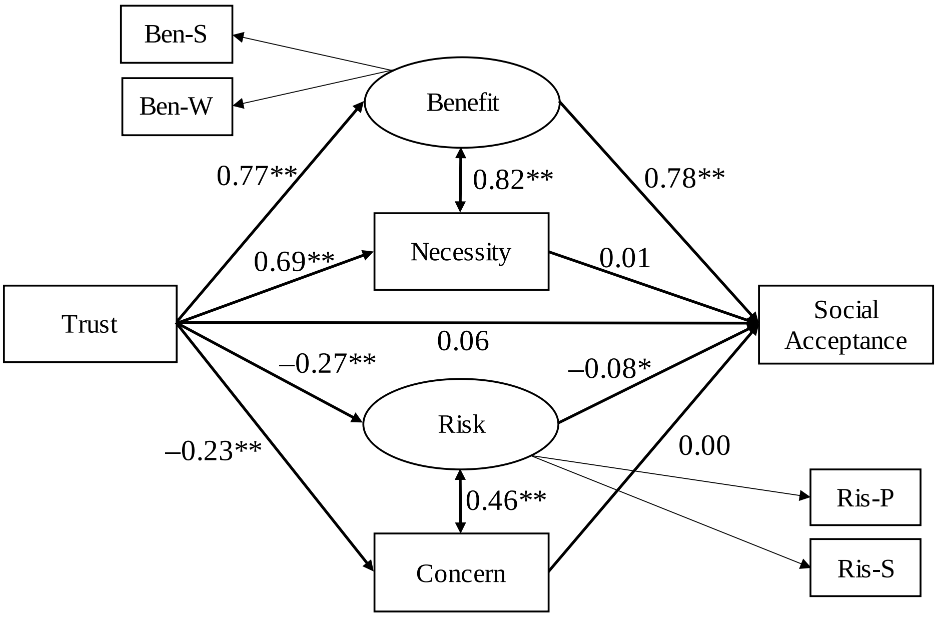

Figure S7. Results of SEM on male participants ($N = 248$). RMSEA = 0.05, AGFI = 0.91, CFI = 0.99. Coefficients are standardized and all coefficients of measurement equations are significant ($\beta > 0.71$, $p < 0.001$). * $p < 0.05$, ** $p < 0.01$.

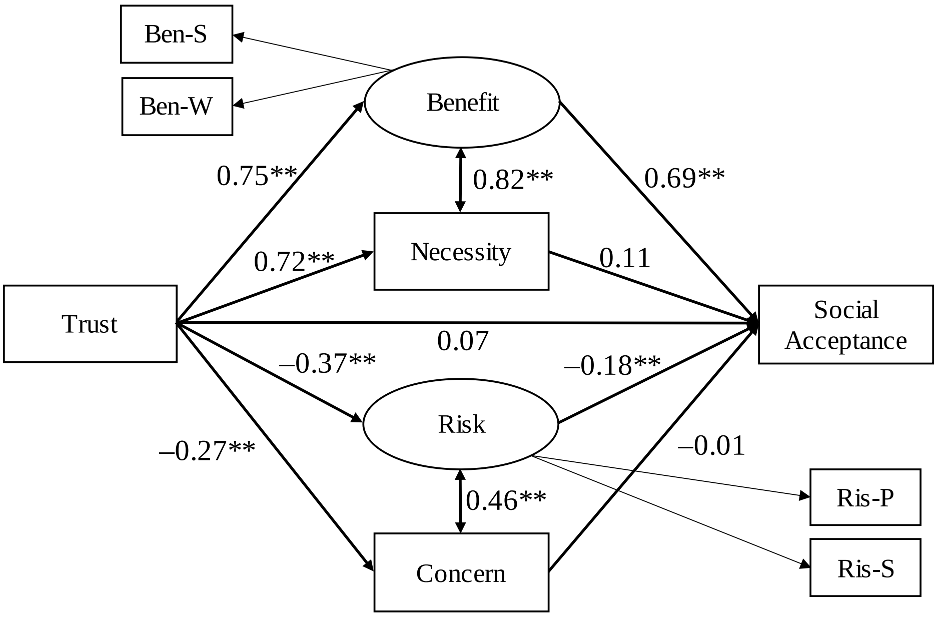

Figure S8. Results of SEM on female participants ($N = 388$). RMSEA = 0.04, AGFI = 0.94, CFI > 0.99. Coefficients are standardized and all coefficients of measurement equations are significant ($\beta > 0.65$, $p < 0.001$). ** $p < 0.01$.

5. Bayesian Structural Equation Modeling

We also analyzed the model by Bayesian SEM (Figure S9), which is less sensitive to sample size. Bayesian SEM can easily estimate more complex models due to technological advances in Markov chain Monte Carlo sampling [1]. Even when we conducted Bayesian SEM, the results were not significantly different from those reported in the main text. The index of fit was: WAIC = 11,418.28, LOOIC = 11,418.44, posterior predictive p (*ppp*) = 0.29. Here, *ppp* values closer to 0.5 indicate that the model fits the observed data, while values closer to 0 indicate less fitness; a threshold of 0.05 is appropriate [1,2].

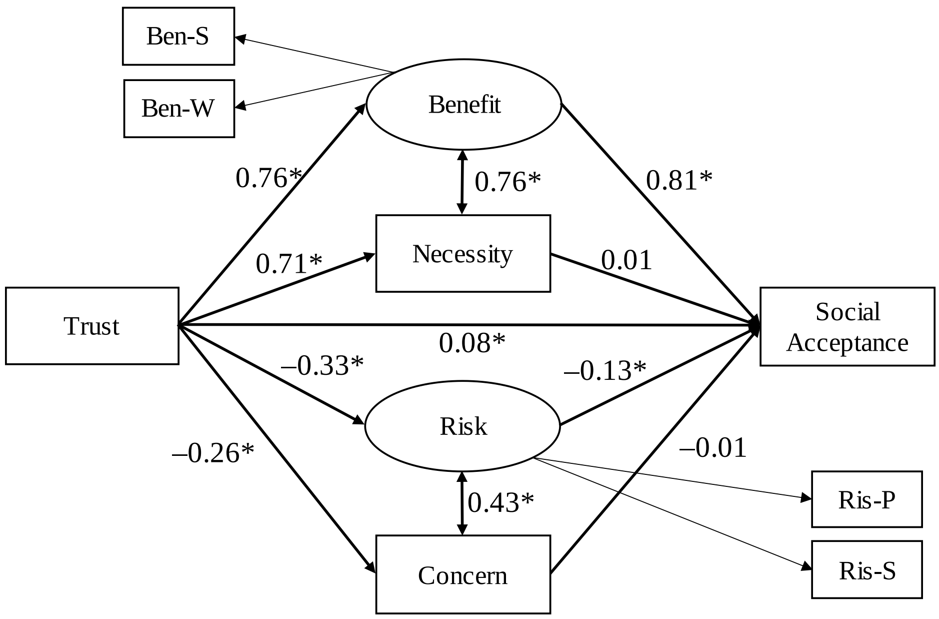

Figure S9. Bayesian SEM results. All coefficients are standardized. * indicates that the 95% credible interval does not straddle zero. The 95% credible intervals of all coefficients of measurement equations do not straddle zero.

References

1. Merkle, E.C.; Rosseel, Y. blavaan: Bayesian structural equation models via parameter expansion. *Journal of Statistical Software*, **2018**, *85*, 1–30, https://doi.org/10.18637/jss.v085.i04.
2. Muthén, B.; Asparouhov, T. Bayesian structural equation modeling: A more flexible representation of substantive theory. *Psychological Methods*, **2012**, *17*, 313–335, https://doi.org/10.1037/a0026802.

6. Mediation Effects

To test the significance of mediation effects, we conducted the method of bootstrapping on the indirect effects in the model ($N_{bootstrap}$ = 2,000). The results showed that, for any of the following mediating variables, the 95% confidence intervals of the estimated effects did not straddle zero (Table S1). The bootstrap method was performed using the statistical software HAD (ver.17; [1]).

Table S1. Results of bootstrapping method.

	**Independent Variable**	**Mediating Variable**	**Dependent Variable**	**Estimated Effect**	**95% CI**
1	Trust	Ben-S	Social acceptance	0.43	[0.38, 0.48]
2		Ben-W		0.30	[0.25, 0.35]
3		Nec		0.45	[0.39, 0.51]
4		Ris-P		0.04	[0.02, 0.06]
5		Ris-S		0.02	[0.01, 0.04]
6		Con		0.03	[0.02, 0.06]

Reference

1. Shimizu, H.; Murayama, A.; Daibo, I. Analyzing the interdependence of group communication (1): Application of hierarchical analysis into communication data. *IEICE Technical Report*, **2006**, *106*, 1–6.
